# Association Between Late-Life Hypertension and Resilience to Alzheimer Dementia Among Older Adults

**DOI:** 10.1093/geroni/igab046.1180

**Published:** 2021-12-17

**Authors:** Mo-kyung Sin, Yan Cheng

**Affiliations:** 1 Seattle University, Seattle, Washington, United States; 2 George Washington University, Washington, District of Columbia, United States

## Abstract

While midlife hypertension is known as one contributing factor for cognitive impairment and Alzheimer dementia in late-life older adults, less is known about the role of late-life hypertension in resilience to Alzheimer dementia. We examined the relationship between late-life hypertension and Alzheimer dementia resilience among older adults using the National Alzheimer’s Coordinating Center data from 2005-2020 (n=3,170). Hypertension, captured within 5 years prior to death, was defined as blood pressure (BP) ≥ 140/90 mmHg in at least two visits and/or ever treated with anti-hypertensive agents. Resilience was defined as positive Alzheimer disease (AD) pathology (CERAD score moderate or severe and BRAAK stage V or VI) from autopsy and Clinical Dementia Rating (CDR) - Sum of Boxes (SOB): 0.5-2.5 or CDR global (0-0.5) from last data point before autopsy. Student’s t-tests and Chi-square tests were conducted to compare patients with and without resilience. A multivariate logistic regression was conducted to estimate the association between late-life hypertension and resilience, adjusting for covariates of demographics and neuropathological characteristics. We had 55 resilient cases among 1,195 positive AD pathology cases. Those resilient were older (88±6.7) and had higher systolic BP (136 ± 18.2 mmHg) than non-resilient (82±7.9 years old, 130±20 mmHg. Untreated hypertension had a protective effect on resilience (adjusted OR: 3.69 (1.10-13.5, p=0.05). Patients with a systolic BP in the range of 135-145 mmHg and a diastolic BP in the range of 65-75 mmHg had the highest resilience possibility. Unlike midlife hypertension, late-life hypertension may have different effect on dementia, prompting further studies.

